# The Validation and Clinical Implementation of BRCAplus: A Comprehensive High-Risk Breast Cancer Diagnostic Assay

**DOI:** 10.1371/journal.pone.0097408

**Published:** 2014-05-15

**Authors:** Hansook Kim Chong, Tao Wang, Hsiao-Mei Lu, Sara Seidler, Hong Lu, Steven Keiles, Elizabeth C. Chao, A. J. Stuenkel, Xiang Li, Aaron M. Elliott

**Affiliations:** 1 Department of Research and Development, Ambry Genetics, Aliso Viejo, California, United States of America; 2 Department of Bioinformatics, Ambry Genetics, Aliso Viejo, California, United States of America; 3 Department of Clinical Genetics, Ambry Genetics, Aliso Viejo, California, United States of America; CNR, Italy

## Abstract

Breast cancer is the most commonly diagnosed cancer in women, with 10% of disease attributed to hereditary factors. Although *BRCA1* and *BRCA2* account for a high percentage of hereditary cases, there are more than 25 susceptibility genes that differentially impact the risk for breast cancer. Traditionally, germline testing for breast cancer was performed by Sanger dideoxy terminator sequencing in a reflexive manner, beginning with *BRCA1* and *BRCA2*. The introduction of next-generation sequencing (NGS) has enabled the simultaneous testing of all genes implicated in breast cancer resulting in diagnostic labs offering large, comprehensive gene panels. However, some physicians prefer to only test for those genes in which established surveillance and treatment protocol exists. The NGS based BRCAplus test utilizes a custom tiled PCR based target enrichment design and bioinformatics pipeline coupled with array comparative genomic hybridization (aCGH) to identify mutations in the six high-risk genes: *BRCA1*, *BRCA2*, *PTEN*, *TP53*, *CDH1*, and *STK11*. Validation of the assay with 250 previously characterized samples resulted in 100% detection of 3,025 known variants and analytical specificity of 99.99%. Analysis of the clinical performance of the first 3,000 BRCAplus samples referred for testing revealed an average coverage greater than 9,000X per target base pair resulting in excellent specificity and the sensitivity to detect low level mosaicism and allele-drop out. The unique design of the assay enabled the detection of pathogenic mutations missed by previous testing. With the abundance of NGS diagnostic tests being released, it is essential that clinicians understand the advantages and limitations of different test designs.

## Introduction

Breast cancer is a complex and multifactorial disease resulting in abnormal cell growth that leads to malignant tumor formation. It is the most common female cancer, affecting one in eight women during their lifetime. Approximately 10% of all breast cancers are hereditary, most commonly caused by genetic variations transmitted in an autosomal dominant manner [1]–[2]. These hereditary breast cancers are typically characterized by early age of onset and bilateral or multiple tumors [3]. The high-risk and well characterized tumor suppressor genes *BRCA1* and *BRCA2* are thought to be responsible for the majority of hereditary breast cancer, with germline mutations conferring a life-time risk of 55–85% and 35–60% respectively [4]–[6]. To date, most individuals with a strong family history of breast cancer are only tested for *BRCA1* and *BRCA2* variants due to well established preventative treatment and surveillance guidelines. However, it is thought more than half of individuals meeting the National Comprehensive Cancer Network (NCCN) criteria for genetic testing do not harbor causative mutations in *BRCA1* or *BRCA2* [7]. Additional susceptibility genes associated with significantly elevated breast cancer risk include *TP53*, *PTEN*, *STK11*, and *CDH1* [8]–[12]. Importantly, similar to *BRCA1* and *BRCA2* these high risk genes are well characterized and medical management guidelines are widely available and well supported to guide preventative and therapeutic decisions based on the results.

The introduction of next-generation sequencing technologies (NGS) has aided in the identification of new cancer susceptibility genes and enabled physicians and patients the option of sequencing multiple genes simultaneously in a cost effective manner [13]–[14]. Several clinical diagnostic laboratories offer multi-gene breast cancer testing ranging from 2–50 genes. However, the addition of newly identified and moderate/low penetrance genes on a test can make the results cumbersome and challenging for a physician to interpret and guide treatment. Therefore, based on the clinician and/or the patient background many physicians prefer to analyze only known high-risk genes in which an established surveillance and treatment protocol exists. Here, we describe the design and validation of BRCAplus, a comprehensive custom clinical diagnostic assay that detects mutations in the six high-risk breast cancer susceptibility genes: *BRCA1*, *BRCA2*, *CDH1*, *PTEN*, *TP53* and *STK11* (Table 1) using NGS and array comparative genomic hybridization (aCGH). This extensive validation illustrates the importance for clinicians and genetic counselors to understand the technologies being used by different laboratories for multi-gene NGS testing, including: target enrichment assay, sequencing technology and bioinformatics pipeline utilized, as all can significantly affect the quality of the results. In addition, the detection of two pathogenic mutations, not previously identified through clinical testing, underscores the sensitivity of our assay and illustrates the importance of second opinion testing.

**Table 1:** High-risk hereditary breast cancer genes analyzed on the BRCAPlus panel.

Gene Symbol	RefSeq ID	Chr.	No. of Exons (Coding Exons)	Gene Length (kb)	Amino Acid Length
BRCA1	NM_007294.3	17q21	23 (22)	81.2	1863
BRCA2	NM_000059.3	13q12.3	27 (26)	84.2	3418
CDH1	NM_004360.3	16q22.1	16	98.2	882
PTEN	NM_000314.4	10q23.3	9	105.3	403
STK11	NM_000455.4	19p13.3	10 (9)	22.6	433
TP53	NM_000546.5	17p13.1	11 (10)	19	393

## Materials and Methods

### DNA Samples

BRCAplus analysis was conducted on 250 previously characterized, archived genomic DNA samples and an additional 3,000 clinical samples sent in for testing. All individuals used for testing provided written consent. All data was de-identified prior to analysis. The study was approved by Solutions Institutional Review Board (protocol #1402060). At least 6∼7 µg of genomic DNA was extracted from whole blood or saliva using the QiaSymphony instrument (Qiagen) according to the manufacturer's instructions. Isolated DNA was quantified using a NanoDrop UV spectrophotometer (Thermo Scientific) and/or Qubit Fluorometer (Life Technologies) with quality metrics of A260/280 = 1.8–2.0. and A260/230>1.6.

### Primer Library Design

A custom primer library was designed using the manufacturer's suggested hybridization parameters (RainDance Technologies). Primers were designed to avoid placement on known SNPs (dbSNP137) and causative gene mutations. Targeted regions that failed to produce PCR amplicons with the standard parameters were re-designed until successful amplification was achieved. Amplicons were designed in a tiling fashion to provide redundancy. Adapter sequences corresponding to a portion of the Illumina NGS adaptor were added to the target-specific portion of the primers. The sequences 5′CGCTCTTCCGATCTCTG3′ and 5′TGCTCTTCCGATCTGAC3′ were added to the forward and reverse primers respectively. The final library consisted of 342 amplicons ranging from 110–199 base pairs (bp) covering all coding exons of the targeted genes and at least 50 bp flanking intronic sequences.

### Target Enrichment and NGS Library Preparation

Five (5) µg of genomic DNA per sample was sonicated to 3–4 kilobase (kb) fragments with the Covaris LE220 instrument following the manufacturer's recommendation. Emulsion PCR droplet reactions were generated and processed using the RainDance Thunderstorm (Raindance Technologies) instrument using the recommended protocol. Samples were collected and PCR amplification performed in a Bio-Rad MyCycler (Bio-Rad) with the following conditions: 94°C for 2 min, followed by a program of 94°C for 30 s, 54°C for 30 s, and 68°C for 15 s for 55 cycles and ending with a 10 min extension at 68°C. To break the emulsion PCR reactions, 70 µl of droplet destabilizer (Raindance Technologies) was added to each sample, vortexed for 15 s and centrifuged at 3200 g for 10 min. Reactions were purified using AMPure XP beads (Beckman Coulter) according to the manufacturer's instructions. A secondary PCR was performed to integrate the remaining sequences of the Illumina adapter and sample-specific barcodes to the amplicons. The sequences of the secondary PCR primers were as follows:

Forward primer:

5′AATGATACGGCGACCACCGAGATCTACAC(*index*)ACACTCTTTCCCTACACGACGCTCTTCCGATCTCTG3′

Reverse primer: 5′CAAGCAGAAGACGGCATACGAGAT(*index*)GTGACTGGAGTTCAGACGTGTGCTCTTCCGATCTGAC3′

For secondary amplification, 10 ng (2.5 ng/µl) of the first PCR reaction was added to 3.25 µl of High-Fidelity Buffer (Invitrogen), .88 µl of MgSO_4_ (Invitrogen), .88 µl of 10 mM dNTP (Invitrogen), 2.5 µl of 4 M Betaine (Sigma), 1.25 µl dimethyl sulfoxide (Sigma), 2.5 µl of 5 µM secondary PCR primers, .5 µl 5 units/µl of Platinum High-Fidelity Taq (Invitrogen), and 9.24 µl of nuclease-free water. Secondary PCR amplification was performed in a Bio-Rad MyCycler (Bio-Rad) with the following conditions: 94°C for 2 min, followed by a program of 94°C for 30 s, 56°C for 30 s, and 68°C for 60 s for 10 cycles and ending with a 10 min extension at 68°C. Reactions are purified using AMPure XP beads (Beckman Coulter) according to the manufacturer's instructions and analyzed using the Fragment Analyzer (Advanced Analytical) or Bioanalyzer instrument (Agilent Technologies). The final libraries were approximately 250–350 bp in size. Libraries are diluted to 10 nM and up to 192 samples pooled together for sequencing. Sequencing was conducted on the Illumina HiSeq2500 using 150 bp paired-end conditions as described in the manufacturer's standard workflow (Illumina).

### Next Generation Sequencing Analysis Parameters

Initial data processing and base calling, including extraction of cluster intensities, was done using RTA 1.17.21.3 (Real Time Analysis, HiSeq Control Software version 2.0.10). Index De-multiplexing was done in the Illumina Consensus Assessment of Sequence and Variation (CASAVA) software (v.1.8.2, Illumina, Hayward, CA). Sequence quality filtering script was also executed in CASAVA. Quality metric data were produced and analyzed through html files including summary.html provided with the data. Data quality was examined in the perfect.html file, which shows the approximate proportion of sequences with 1, 2, 3 or 4 errors. Other quality metrics included IVC plots, visualizations of cluster intensity over the duration of the sequencing run. The summary.html field “Lane Yield” was calculated as the number of sequence clusters that pass quality filters (PF, this value is also displayed as % PF Clusters) multiplied by the number of tiles per lane (120) multiplied by the number of sequencing cycles.

The custom NGS bioinformatics pipeline utilized Novoalign V3.00.05 to align FASTQ reads to a reference sequence and utilized GATK V2013.1-2.4.9 to generate variant and no/low coverage reports. Bioinformatic NGS pipeline later filtered variants with a Q score ≥20 and an allele count ≥50X, along with no/low coverage regions that are ≤50X coverage.

### NGS Data Analysis and Interpretation

During variant calling, bases corresponding to the forward and reverse primers were trimmed off using internally developed custom ‘primer trimming’ script. After trimming, all the trimmed bases were marked as ‘soft-clipped’ and the alignment information reconstructed. The newly trimmed BAM output file was used as an input for the subsequent variant calling by GATK V2013.1-2.4.9.Variants of each sample within the reportable range (coding exons plus at least 5 bases of flanking intronic sequence) are filtered out if determined to be a sequencing artifact or common polymorphism utilizing population frequency data from multiple sources including NCBI dbSNP, NHLBI Exome Sequencing Project (ESP), 1000 Genomes, and internal Ambry data. Known causative variants outside reportable range are also protected from filtering. Sanger sequencing is utilized to confirm all variants and cover any regions with insufficient read depth. Variants are then classified as pathogenic in accordance with recommendations from the American College of Medical Genetics or based on the scientific literature [15]–[16]. Sequencing variant data was deposited into the publically available ClinVar (https://www.ncbi.nlm.nih.gov/clinvar/) and Breast Cancer Information Core (BIC) (http://research.nhgri.nih.gov/bic/) databases (due to the large amount of accession numbers, accession numbers can be requested from the Corresponding Author).

### Array Design

A custom microarray with exon level resolution was developed to identify gross deletions or duplications. The microarray was developed using the online application eArray software (Agilent Technologies, https://earray.chem.agilent.com/earray/). The microarray contains approximately 60,000 interrogating oligonucleotide probes that were annotated against the human genome assembly build 37 (February 2009; NCBI37/hg19). Probes density was increased in exons and 300 bp flanking intronic sequence, with an average of 13 probes/exon. In addition, probes were placed every 2.5 Kb of intronic sequence and heavily tiled in promoter regions. Following validation runs only those probes with optimal performance were selected for the final array design.

### Array Comparative Genomic Hybridization (aCGH)

The procedures for DNA digestion, labeling, and hybridization for the oligo arrays were performed according to the standard Agilent protocol v7.1, with minor modifications. Briefly, 0.25 µg of patient genomic DNA and 0.25 µg of pooled gender-matched reference DNA (Promega) were labeled with Cyanine 5 (Cy5) or Cyanine 3 (Cy3) dyes (Agilent). Following purification with Amicon 30 kDa filters (Millipore), the labeled DNA yield and dye incorporation were measured using an ND-2000 spectrophotometer (NanoDrop). The Cy3-and Cy5-labeled samples along with 2 µg human Cot-1 DNA (Invitrogen), 10X blocking agent (Agilent) and 2X Hi-RPM Buffer (Agilent) were added and hybridized together at 65°C on the CancerArray (Ambry Genetics) for 24 h in a rotisserie oven at 20 rpm. Slides were washed according to the manufacture's protocol and scanned at 3 µm resolution on an Agilent G2565CA high-resolution DNA microarray scanner.

#### aCGH Data Analysis

Data was extracted using Agilent Feature Extraction software (version 11.0.1.1) using the CGH_1100_Jul11 protocol, then analyzed for copy-number changes using Agilent Genomic Workbench 7.0 software package (Agilent Technologies, CA) and/or BioDiscovery Nexus 6.1 (BioDiscovery).

To correct for GC content, a noise reducing systematic correction file was developed based on the genomic locations of the probes in the design. Log2 ratios were computed and normalized by the centralization algorithm in Genomic Workbench. The fuzzy zero correction was applied to remove putative variant intervals with small average log2 ratios between probes in long genomic intervals. For Genomic Workbench, aberrant regions were determined by the Aberration Detection Method-2 (ADM-2) algorithm with a threshold of 6.0. For Nexus, aberrant regions were determined using the FASST2 Segmentation algorithm with a significance threshold of 1.0E-5. The aberration filter was selected with the following parameters: minimum number of probes in the region 4, minimum absolute average log_2_ ratio for one copy amplification was .35 and for a heterozygous deletion -.45, and a mean log_2_ ratio >.6 represents a high copy gain and <-1 a homozygous copy loss. Causative abnormalities were confirmed using Multiplex Ligation-Dependent Probe Amplification (MLPA) Analysis (MRC-Holland).

## Results

### Assay Design and Workflow for BRCAplus Test

**Figure 1:**
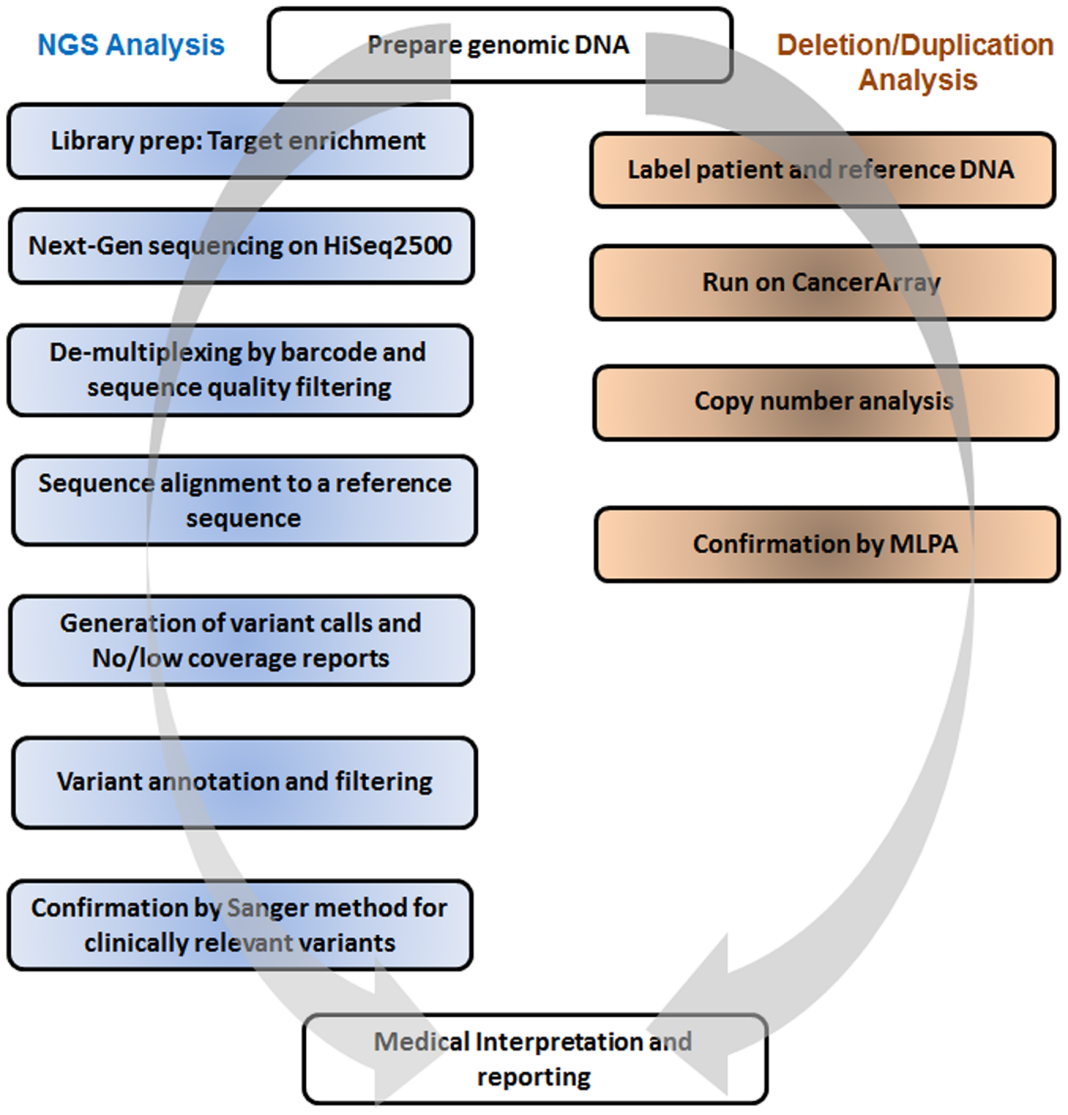
Schematic diagram of BRCAplus assay workflow.

The custom targeted BRCAplus test includes comprehensive analyses of all 92 coding exons in six high-risk breast cancer susceptibility genes: *BRCA1*, *BRCA2*, *CDH1*, *PTEN*, *TP53*, and *STK11* (Table 1). Using a combination of NGS and aCGH, the assay is designed to detect nucleotide substitutions, small deletions, small insertions, small indels, and gross deletions/duplications. To essentially eliminate false positives, all reportable NGS variants were confirmed using Sanger dideoxy chain termination sequencing. Similarly, detected aCGH abnormalities were verified using MLPA technology, when applicable (Figure 1).

There are several methods one can use for primer design of a diagnostic assay. Due to the short read lengths of NGS, most labs employ a method of PCR based target enrichment followed by amplicon concatenation, sonication and adapter ligation. This method is costly, time consuming and results in only ∼30–40% of reads on target due to the high percentage of chimeric amplicons (internal data) [17]. To eliminate the need for NGS library preparation and increase target specificity, primers were designed to include a portion of the NGS adapter on the tail of the target specific primer. Following a secondary PCR to incorporate the remaining adapter sequence and sample barcode, the amplicons are ready to sequence.

**Figure 2:**
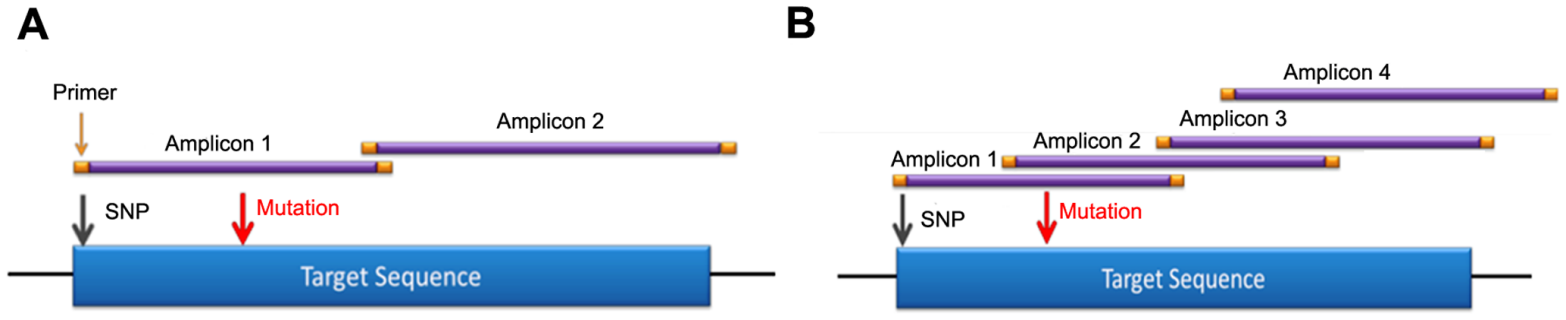
Redundant primer tiling design limits allele drop-out. A) A typical primer design with one to two amplicons covering region of interest. A polymorphism under the primer of amplicon one would result in allele drop-out and a false negative. B) BRCAplus tiling primer design has overlapping amplicons designed over region of interest. The same polymorphism in amplicon one would not result in false negative due to amplicon redundancy of amplicon two.

**Figure 3:**
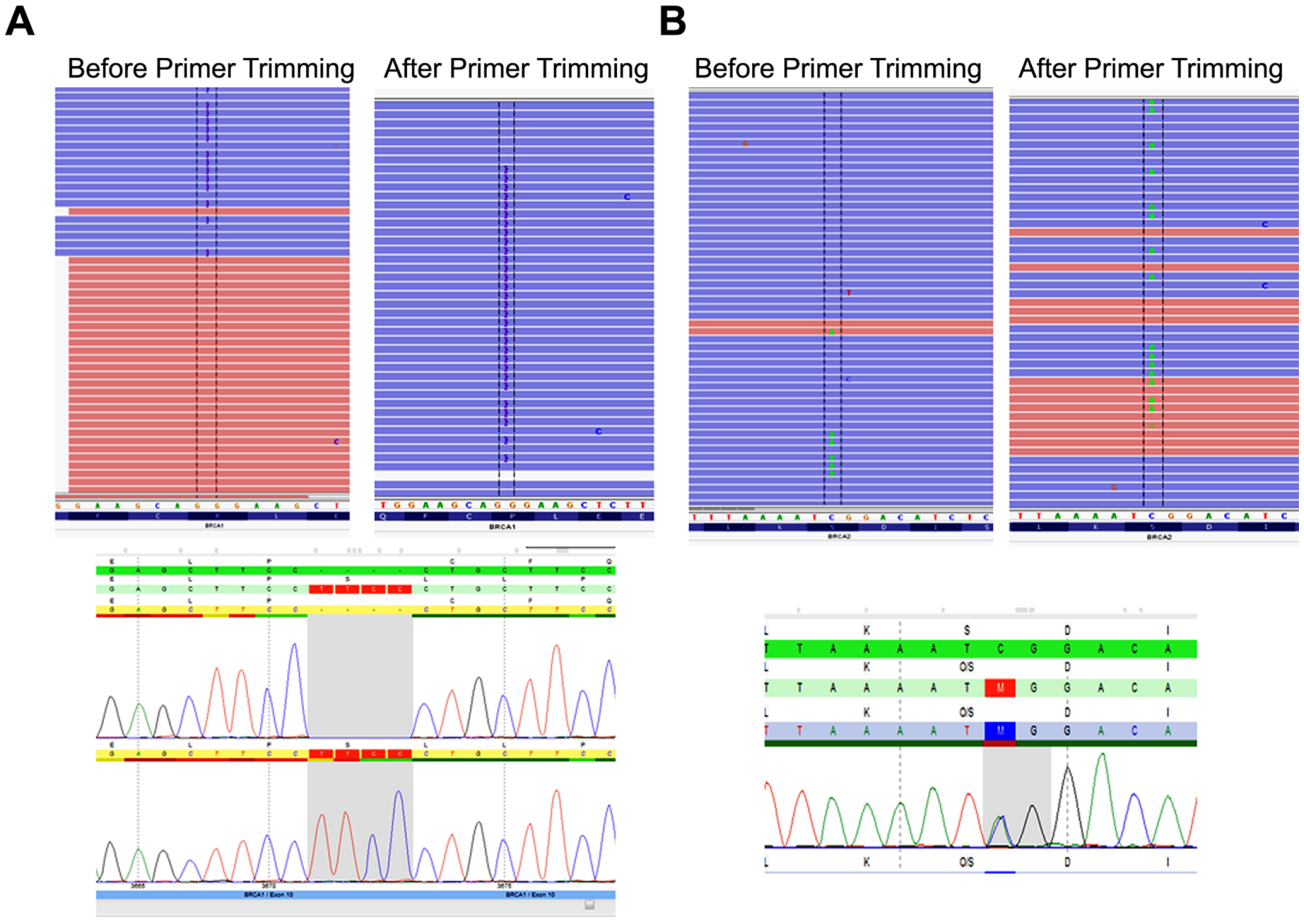
Primer sequence trimming increases detection sensitivity. Two heterozygous causative variants A) *BRCA1* c.3671_3672insCTTC and B) *BRCA2* c.2918C>A that were missed without primer trimming were detected with the function enabled in the pipeline. Both calls were confirmed with Sanger sequencing.

Primer based target enrichment techniques used in Sanger sequencing and NGS often produce false negatives due to polymorphisms located under primer binding sequences, which interrupts primer hybridization resulting in amplicon dropout [18]–[19]. If the polymorphism occurs on the same allele as the causative mutation, the mutation will go undetected. The custom primer tiling design of the BRCAplus test limits allele drop-out due to the placement of overlapping, redundant amplicons covering the target sequences. This ensures an exceedingly small chance of false negatives due to rare variants or polymorphisms under a primer binding site (Figure 2A and 2B). Importantly, since the primer sequences themselves are sequenced and included in the data, it is essential that the primer sequence data is not included in the analysis, as it has the potential to dilute out the actual DNA sequence under the primer sites, decreasing assay sensitivity in the regions covered by the PCR primers. To address this problem, the primer sequences for each read were trimmed off during variant calling as described in the Materials and Methods section. To test the validity of primer trimming, we compared the variant data from 3,000 BRCAplus patient samples before and after primer trimming. We were able to detect two additional causative mutations (*BRCA1* c. 3671_3672insCTTC and *BRCA2* c.2918C>A) that were not identified without the addition of the primer trimming function. The percent of mutant reads for the heterozygous *BRCA1* c.3671_3672insCTTC frameshift mutation and *BRCA2* c.2918C>A nonsense mutation increased from 11% to 44% and 8% to 45% respectively, crossing the threshold for detection of the disease-causing mutations by our bioinformatics pipeline (Figure 3A and 3B).

To avoid the limitations of calling gross abnormalities off NGS data we utilized a custom targeted microarray with exon level resolution to detect copy number variations in the genes of interest [20]. The microarray contains an average of 13 probes per exon, with additional probes spaced every 2.5 Kb in the intronic regions.

### Analytical Validity of BRCAplus Diagnostic Assay

To test the analytical sensitivity and accuracy of the BRCAplus assay we sequenced peripheral blood and saliva DNA from 250 samples. There was an average of 0.36 gigabases (Gb) of high quality (>Q20) sequence per sample (range 0.18 to 0.56 Gb). On average, 87% of sequence reads were on target, mapping specifically to the intended targeted regions. This resulted in an extremely high mean read depth per nucleotide across all samples of more than 9,000X.

**Figure 4:**
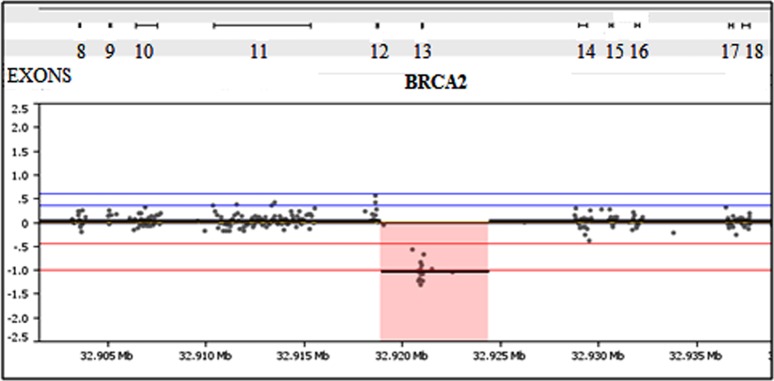
Microarray has exon level resolution for deletion and duplication variant detection. A sample harboring a 2 Kb exon 13 deletion in *BRCA2* was detected using the custom designed microarray.

The 250 samples harbored a total of 3,025 previously defined germline variants in the 6 targeted genes. These samples represented all different types of variants the test is designed to detect including synonymous, missense, nonsense, splice-site, small duplications and deletions and gross deletions and duplication abnormalities. The BRCAplus test correctly identified all variants, including causative mutations concordant with previous testing, resulting in 100% sensitivity [True Positives/(True Positives + False Negatives)] (Table 2). Importantly, these detected abnormalities include single exon deletions, which illustrates the sensitivity and accuracy of the custom targeted microarray (Figure 4).

**Table 2:** Validation of BRCAplus deleterious variant detection on previously characterized samples.

Sample ID	Gene	Coding Variant	Protein Variant	HET/HOMO
50103	BRCA2	c.4965C>G	p.Y1655*	HET
53997	BRCA2	c.3922G>T	p.E1308*	HET
56169	BRCA2	c.658_659delGT	p.V220Ifs*4	HET
56413	BRCA1	c.4508C>A	pS1503X	HET
56800	BRCA1	c.4524G>A	p.W1508X	HET
59648	BRCA2	c.7069_7070delCT	p.L2357Vfs*2	HET
59648	TP53	c.88_90delAAC	No change	HET
59703	BRCA2	c.7558C>T	p.R2520*	HET
60599	BRCA2	c.9026_9030delATCAT	p.Y3009Sfs*7	HET
63266	BRCA1	c.5193+1G>C	Splice variant	HET
66230	PTEN	c.74dupT	p.L25Ffs*19	HET
67019	STK11	c.250A>T	p.K84*	HET
67126	TP53	c.1010G>A	p.R337H	HET
67802	TP53	c.818G>A	p.R273H	HET
70317	STK11	c.1211C>T	p.S404F	HET
73031	STK11	c.297T>G	p.I99M	HET
73805	CDH1	c.1531C>T	p.Q511*	HET
74802	PTEN	c.422A>G	p.H141R	HET
77388	STK11	c.106delT	p.Y36Tfs*15	HET
77914	PTEN	c.377C>T	p.A126V	HET
79755	CDH1	c.1237_1238dupTA	p.I415Pfs*3	HET
NA13708	BRCA1	c.4689C>G	p.Y1563*	HET
NA13709	BRCA1	c.2071delA	p.R691Dfs*10	HET
NA14090	BRCA1	c.70_71delAG	p.C24Sfs*16	HET
NA14622	BRCA2	c.6275_6276delTT	p.L2092Pfs*7	HET
NA14622	BRCA2	c.9976A>T	p.K3326*	HET
NA14623	BRCA2	c.125A>G	p.Y42C	HET
NA14624	BRCA2	c.5722_5723delCT	p.L1908Rfs*2	HET
NA14626	BRCA2	c.9976A>T	p.K3326*	HET
NA14637	BRCA1	c.4327C>T	p.R1443*	HET
NA14639	BRCA2	c.6198_6199delTT	p.V2066Vfs*11	HET
61720	TP53	5UTR_ex11del	25.6kb Del	HET
60103	CDH1	ex3del	6.9kb Del	HET
S0008	TP53	Ex2-6 Del	4.1kb Del	HET
91412	BRCA2	Ex13Del	2.0kb Del	HET

Table illustrates representative calls from 250 accuracy samples. All mutations confirmed by Sanger or MLPA.

HET, heterozygous; *, Stop codon; fs, frame-shift; del, deletion; dup, insertion; CNV, copy number variation.

In a high-throughput diagnostic lab, it is essential to limit the number of false positive NGS variants that need Sanger confirmation due to the associated time and labor involved. To determine the false positive rate of the BRCAplus assay, we analyzed the total 23,153 base pair reportable range for the 250 previously characterized samples. There were a total of 30 false positives from 5,788,250 base pairs interrogated, resulting in an NGS analytical specificity of 99.99% [True Calls/(True Calls + False Positives)]. Several of the false positives identified were redundant, with only 14 unique calls. Clearly, any test which Sanger confirms NGS variants has an overall analytical specificity of 100%.

### BRCAplus in the Clinic

**Figure 5:**
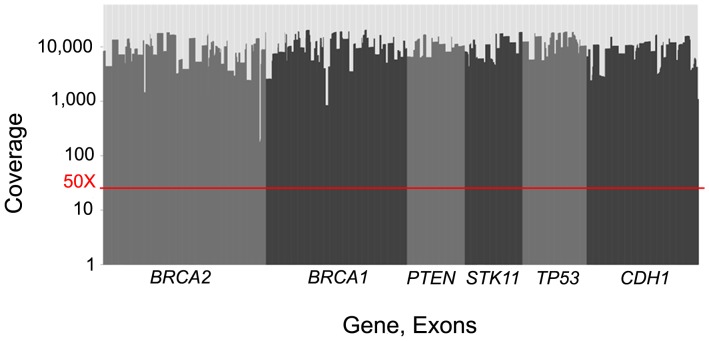
Average depth of coverage for each exon on the test from 3,000 representative samples. The red line indicates the 50X coverage threshold in which any region with insufficient coverage would be Sanger sequenced.

To demonstrate the performance of the BRCAplus test in the clinic we analyzed the data from the first 3,000 patient samples referred to Ambry Genetics for testing. Due to the relatively small size of the genomic loci interrogated, up to 192 barcoded samples were pooled and sequenced per lane on the Illumina HiSeq2500 instrument. The average sequencing coverage per nucleotide was 9,717X (Figure 5).

Although rare, to ensure every base pair in the targeted regions are sequenced with sufficient coverage, any base pair under 50X coverage is flagged in the bioinformatics pipeline and Sanger sequenced.

**Figure 6:**
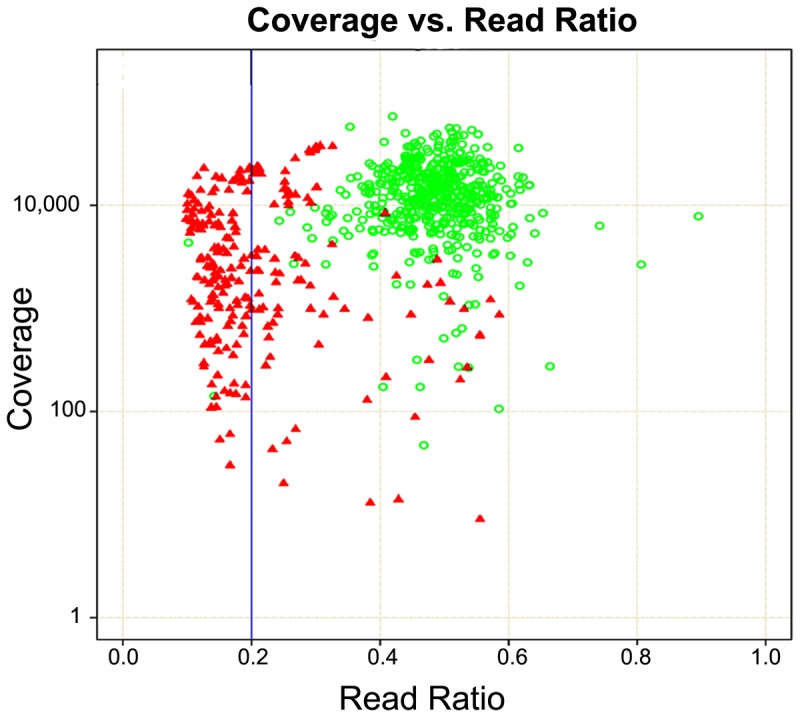
Heterozygous read ratio vs. read coverage. To identify the profile of false positives, sequencing coverage was plotted against the heterozygous read ratios using Sanger sequencing confirmed NGS variants. Green circle, Sanger confirmed variants. Red triangle, Sanger cleared false positive.

Analyzing the data from all the NGS variant calls and the corresponding Sanger sequencing verifications, there were a total of 354 false positives. More than 99% of false positives identified by NGS had a heterozygous read ratio less than 0.2 and typically lower sequencing coverage than Sanger confirmed variants (Figure 6). However, some false positives had similar read ratios and sequencing coverage as true calls. Conversely, two true causative variants had a read ratio below 0.2 mimicking a false positive.

**Figure 7:**
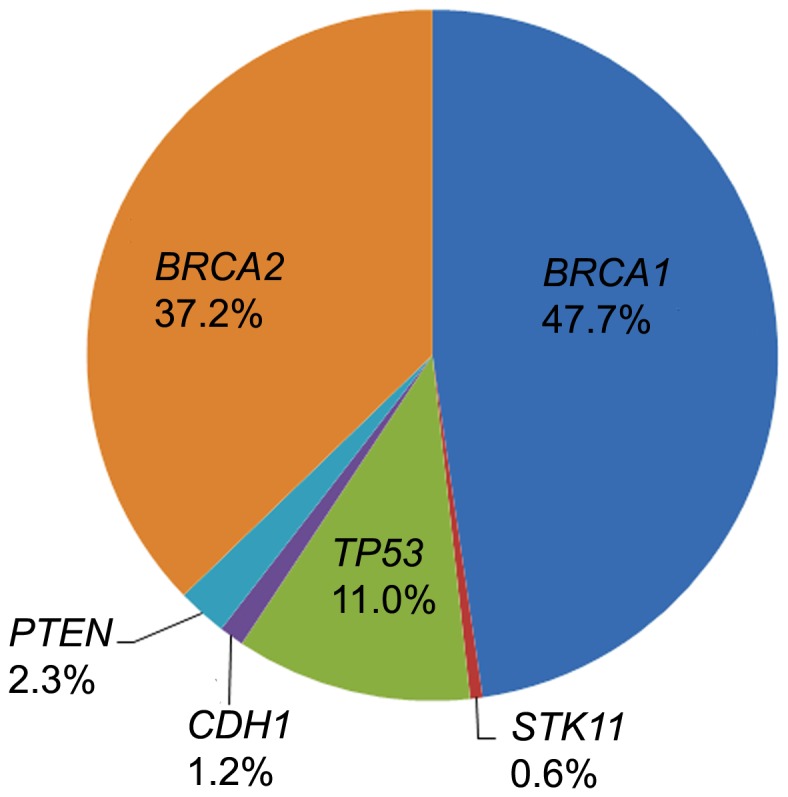
Distribution of pathogenic mutations reported out in first 3,000 patient samples referred for BRCAplus testing.

**Figure 8:**
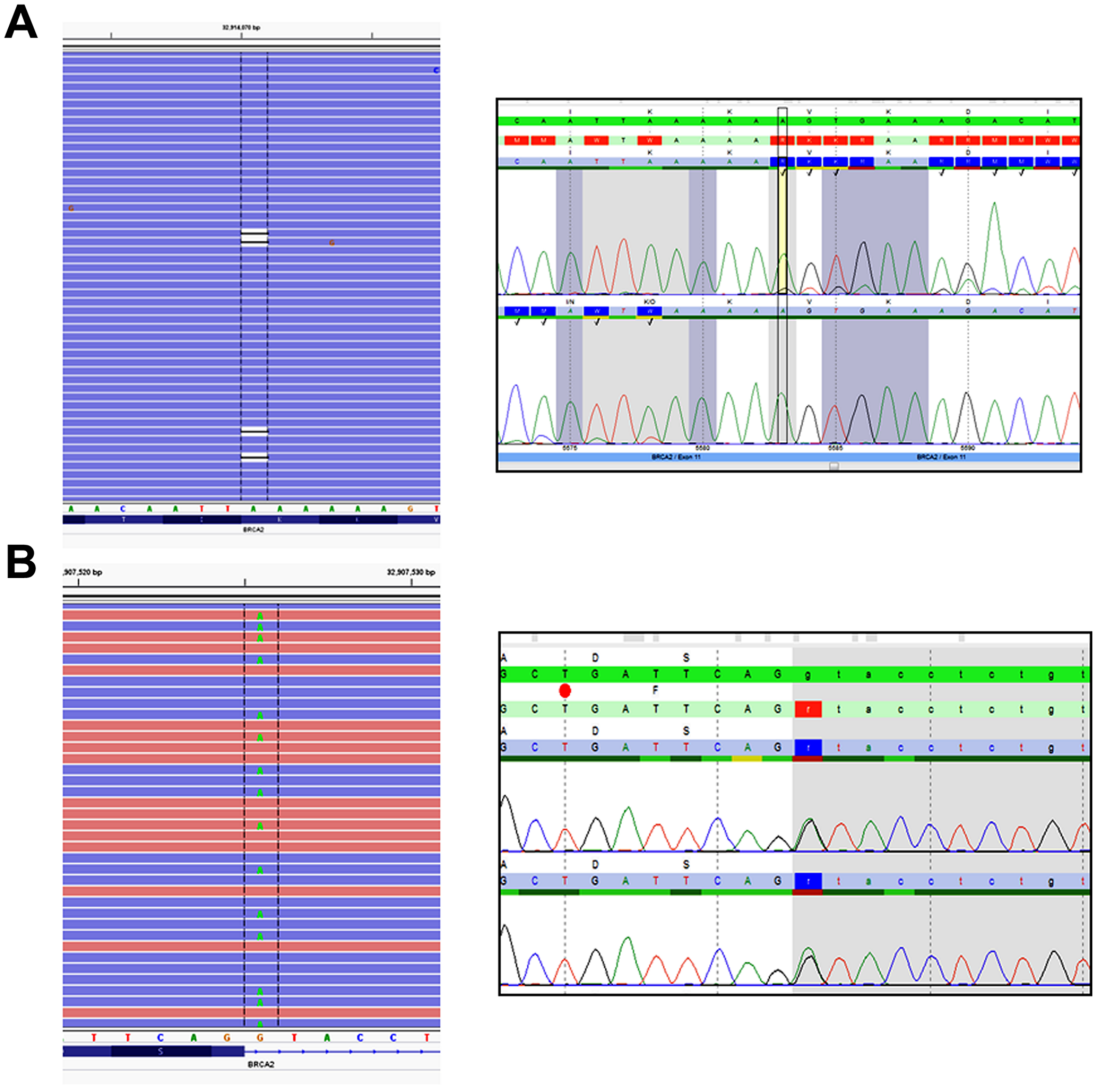
Causative heterozygous mutations, which had previously gone undetected, identified with BRCAplus assay. A) Mosaic pathogenic mutation c.5583delA in *BRCA2* was detected at allele ratio of 14.9% and confirmed by Sanger sequencing. B) A heterozygous splice site mutation c.1909+1 G>A in *BRCA2*, was detected by BRCAplus assay.

In the first 3,000 patient samples who were referred to Ambry Genetics for high-risk BRCAplus testing, 172 were positive for a deleterious mutation (5.7%), 228 had inconclusive results (7.6%) and 2,600 were negative (86.7%). The *BRCA1* and *BRCA2* genes accounted for ∼85% of the positives, while *CDH1*, *PTEN*, *STK11*, and *TP53* accounted for the remaining ∼15% (Figure 7). Notably, some of the patient samples received were sent in for additional testing after having previously tested negative for inherited mutations in *BRCA1* and *BRCA2*. Our methodology identified a pathogenic mosaic *BRCA2* mutation in a patient which was previously undetected by Sanger sequencing. The *BRCA2* c.5583delA mutation was present at an allele frequency of 14.9%, with coverage of 7,439X (Figure 8A). As expected, Sanger sequencing confirmation of the mutation revealed a low level peak on the chromatogram.

As discussed previously, the tiled, redundant primer design of the BRCAplus assay minimizes the chance of allele drop-out and potential false negatives, which is an inherent limitation of sequencing tests which rely on one or two amplicons to cover the entire region of interest. A patient diagnosed with ovarian cancer in her 4th decade and a strong family history of breast and ovarian cancer had previously tested negative for deleterious *BRCA1* and *BRCA2* mutations by another commercial lab. The BRCAplus assay revealed a heterozygous splice-site mutation, *BRCA2* c.1909+1G>A, with coverage of 2,520X and variant frequency of 48% (Figure 8B). Sanger sequencing confirmation verified the germline mutation. These two examples highlight the sensitivity and advantages of the BRCAplus test design in a diagnostic setting and its ability to detect mutations previously missed.

## Discussion

Here, we have described the design, validation, and clinical implementation of a new comprehensive genetic testing option, termed BRCAplus that analyzes the 6 known high-risk breast cancer genes. The test utilizes a unique target enrichment strategy and bioinformatics pipeline for NGS and a custom aCGH platform. With the introduction and quick adoption of NGS technology in diagnostics, clinicians for the first time can sequence all genes implicated in breast/ovarian cancer in one test. However, depending on the circumstances and patient, more genes on a test does not necessarily equal a better test. Only a small number of breast susceptibility genes known today are ‘actionable’ in terms of disease management. There is limited evidence to support analysis of genes other than those for which preventive and therapeutic decisions can be made. The six genes on this test all have well-established lifetime cancer risks, ranging from 30-90% associated with mutations. Depending on the patient and the gene involved, detection of pathogenic variants can support recommendations for increased surveillance or preventative prophylactic surgeries. In addition, cancer therapeutic selection may be based on which gene was found mutated in the test. For example, studies suggest *BRCA1*, *BRCA2*, and *PTEN* mutation carriers may respond well to PARP inhibitors [21]–[23]. Adjuvant tamoxifen treatment may be another option to decrease the risk of cancer occurrence for individuals with *BRCA1* and *BRCA2* mutations who decide not to undergo preventative surgery [24]. The test results could also influence a clinician on how not to treat the patient. Patients harboring a TP53 mutation have an abnormal response to therapeutic radiation resulting in increased risk for secondary tumors and thus this approach should be avoided [25].

The use of NGS in the clinic has revolutionized diagnostic testing, however it has also resulted in a more complicated commercial market from which clinicians and genetic counselors have to try to select the best option for their patients. All tests utilizing NGS are not created equal. Most NGS tests on the market use different target enrichment strategies, NGS platform, or bioinformatics pipeline for data analysis. Labs may choose different methods based on sample volume, turn-around-time, cost or experience. It is important for clinicians ordering NGS tests to realize that depending on the test design there can be serious differences in quality and the types of mutations identified. Our data presented here reaffirms the importance of Sanger confirming all NGS detected variants before clinical reporting out. Other examples include tests using probe based target enrichment which have trouble detecting mutations in regions with pseudogenes or high GC rich regions [26]. In addition, allelic bias can be an issue with probe based target enrichment resulting in low level calls and missed mutations. As shown in our results, assays with non-redundancy or target amplicons with few primer sets, such as those found in Sanger sequencing, can be plagued by allele-drop out. Labs using semiconductor NGS, which utilizes flow based chemistry, will have a difficult time accurately detecting insertions and deletions in homopolymer regions [27]. Arguably, the bioinformatics and software employed for data analysis introduces the most variability in accuracy and sensitivity between different diagnostic NGS tests. It is important that the bioinformatics pipeline be tailored for each gene on a test and account for the methods used for enrichment and sequencing. For example, our data illustrates that without incorporating primer trimming into our pipeline we would have missed two causative mutations in the data set. Diagnostic labs offering testing without extensive bioinformatics support and experience will undoubtedly miss causative variations. Although the detection limit of Sanger sequencing is typically referenced at 10%, it can be highly variable depending on the sequence being analyzed and the performing lab. Unless notified of the mutation beforehand, low level mosaicism is not typically detectable by Sanger sequencing.

Having used NGS since its inception and being the first diagnostic lab to offer hereditary cancer based NGS testing, we were able to leverage our extensive experience in target enrichment and bioinformatics to develop and validate a highly accurate and sensitive assay with a turn-around amenable to clinical use. The design and streamlined workflow has enabled a rapid return of results in10–21 days, which is essential in genetic testing for inherited breast cancer susceptibility where results influence medical management including surgical or treatment decisions. The positive detection rate of the test in the first 3,000 clinical patients described here is influenced by numerous factors. First, patients referred for testing were not stratified according to risk and NCCN guidelines. Also, patients may have already tested negative by another commercial lab for causative variants in one or more of the genes on the test, such as *BRCA1* and *BRCA2*. Likewise, the number of patients in which a causative *STK11* mutation was detected is low; in over 7,500 cases, all mutations in *STK11* were identified in individuals who met clinical criteria for Peutz-Jegher polyposis. Clinicians can typically diagnose individuals harboring mutations in *STK11* due to the unique hallmark presence of mucocutaneous hyperpigmentation and gastrointestinal manifestations. Therefore, only *STK11* gene testing is generally ordered and the diagnostic yield is high. Importantly, this does not appear to be true for patients with mutations detected in *CDH1*, *PTEN*, or *TP53* suggesting that further study is required to explore expanding phenotypes in syndromes associated with these genes.

In conclusion, we have designed and validated a comprehensive, clinically actionable test for high-risk hereditary breast cancer. The test was able to detect all previously characterized variations as well as detect mutations missed by previous testing. The streamlined workflow and test design will enable clinicians to rapidly receive high quality, accurate, clinically meaningful data to aid in their treatment decisions.
