# Correction: The Validation and Clinical Implementation of BRCAplus: A Comprehensive High-Risk Breast Cancer Diagnostic Assay

**DOI:** 10.1371/journal.pone.0110156

**Published:** 2014-09-26

**Authors:** The PLOS ONE Staff

There is an error in Table 2. The word “deleterious” should be omitted from the table title. Please see the corrected Table 2 here.

**Table 2:** Validation of BRCAplus variant detection on previously characterized samples.

Sample ID	Gene	Coding Variant	Protein Variant	HET/HOMO
50103	BRCA2	c.4965C>G	p.Y1655*	HET
53997	BRCA2	c.3922G>T	p.E1308*	HET
56169	BRCA2	c.658_659delGT	p.V220Ifs*4	HET
56413	BRCA1	c.4508C>A	pS1503X	HET
56800	BRCA1	c.4524G>A	p.W1508X	HET
59648	BRCA2	c.7069_7070delCT	p.L2357Vfs*2	HET
59648	TP53	c.88_90delAAC	No change	HET
59703	BRCA2	c.7558C>T	p.R2520*	HET
60599	BRCA2	c.9026_9030delATCAT	p.Y3009Sfs*7	HET
63266	BRCA1	c.5193+1G>C	Splice variant	HET
66230	PTEN	c.74dupT	p.L25Ffs*19	HET
67019	STK11	c.250A>T	p.K84*	HET
67126	TP53	c.1010G>A	p.R337H	HET
67802	TP53	c.818G>A	p.R273H	HET
70317	STK11	c.1211C>T	p.S404F	HET
73031	STK11	c.297T>G	p.I99M	HET
73805	CDH1	c.1531C>T	p.Q511*	HET
74802	PTEN	c.422A>G	p.H141R	HET
77388	STK11	c.106delT	p.Y36Tfs*15	HET
77914	PTEN	c.377C>T	p.A126V	HET
79755	CDH1	c.1237_1238dupTA	p.I415Pfs*3	HET
NA13708	BRCA1	c.4689C>G	p.Y1563*	HET
NA13709	BRCA1	c.2071delA	p.R691Dfs*10	HET
NA14090	BRCA1	c.70_71delAG	p.C24Sfs*16	HET
NA14622	BRCA2	c.6275_6276delTT	p.L2092Pfs*7	HET
NA14622	BRCA2	c.9976A>T	p.K3326*	HET
NA14623	BRCA2	c.125A>G	p.Y42C	HET
NA14624	BRCA2	c.5722_5723delCT	p.L1908Rfs*2	HET
NA14626	BRCA2	c.9976A>T	p.K3326*	HET
NA14637	BRCA1	c.4327C>T	p.R1443*	HET
NA14639	BRCA2	c.6198_6199delTT	p.V2066Vfs*11	HET
61720	TP53	5UTR_ex11del	25.6kb Del	HET
60103	CDH1	ex3del	6.9kb Del	HET
S0008	TP53	Ex2-6 Del	4.1kb Del	HET
91412	BRCA2	Ex13Del	2.0kb Del	HET

Table illustrates representative calls from 250 accuracy samples. All mutations confirmed by Sanger or MLPA.

HET, heterozygous;

*, Stop codon; fs, frame-shift; del, deletion; dup, insertion; CNV, copy number variation.
